# Anxiety in cancer patients

**DOI:** 10.1054/bjoc.2000.1405

**Published:** 2000-10-26

**Authors:** D P H Stark, A House

**Affiliations:** ICRF Department of Medical Oncology, St James's University Hospital, Beckett Street, Leeds, LS9 7TF; Academic Unit of Psychiatry and Behavioural Sciences, 15 Hyde Terrace, Leeds, LS2 9LT, UK

**Keywords:** anxiety, cancer, reassurance

## Abstract

Anxiety is common in cancer patient populations, and must often initially be recognized and managed by cancer care professionals. This article reviews the recent oncology and mental health literature on anxiety. The aim is to help those involved in cancer patient care who are not specialists in mental health to understand the nature of anxiety, and discriminate morbid from normal anxiety. We review recent research into the association of anxiety with events during diagnosis and management of cancer, highlighting the importance of the meaning of events to an individual as an important factor in making people anxious. Lastly we review management strategies which might be used by cancer care professionals, in particular the importance of an awareness of specific patterns of communication which may alleviate or maintain anxiety for some cancer patients. © 2000 Cancer Research Campaign


*British Journal of Cancer* (2000) **83**(10), 1261–1267
© 2000 Cancer Research Campaign
doi: 10.1054/ bjoc.2000.1405, available online at http://www.idealibrary.com on IDEAL®

Review

### Anxiety in cancer patients

**DPH Stark[1] and A House[2]**

[1]ICRF Department of Medical Oncology, St James's University Hospital, Beckett Street, Leeds LS9 7TF; [2]Academic Unit of Psychiatry and Behavioural Sciences, 15 Hyde Terrace, Leeds LS2 9LT, UK

**Summary** Anxiety is common in cancer patient populations, and must often initially be recognized and managed by cancer care professionals. This article reviews the recent oncology and mental health literature on anxiety. The aim is to help those involved in cancer patient care who are not specialists in mental health to understand the nature of anxiety, and discriminate morbid from normal anxiety. We review recent research into the association of anxiety with events during diagnosis and management of cancer, highlighting the importance of the meaning of events to an individual as an important factor in making people anxious. Lastly we review management strategies which might be used by cancer care professionals, in particular the importance of an awareness of specific patterns of communication which may alleviate or maintain anxiety for some cancer patients. © 2000 Cancer Research Campaign

**Keywords**: anxiety; cancer; reassurance

Cancer is threatening, and understandably many patients are anxious in response to that threat. Unfortunately that anxiety sometimes becomes a clinically important problem in its own right, and cancer care professionals will often be responsible for its initial recognition and management. Anxiety does not feature much in the standard oncology literature. In this article we therefore review recent research into anxiety complicating cancer and other physical illnesses.

### THE NATURE OF ABNORMAL ANXIETY IN CANCER

Anxiety produces a number of typical symptoms and signs. Symptoms of autonomic over-activity include palpitation and sweating. Anxious behaviours such as restlessness and reassurance-seeking are a feature. Changes in thinking include apprehension, worry and poor concentration, and physical symptoms such as muscle tension or fatigue may occur.

Since anxiety is a frequent response to threat, it is found in all clinical populations. It can be adaptive, but in certain circumstances it becomes maladaptive or morbid. Such pathological anxiety is identified by:

1. Being out of proportion to the level of threat
2. Persistence or deterioration without intervention
3. A level of symptoms which are unacceptable regardless of the level of threat (these include recurring panic attacks, severe physical symptoms, and abnormal beliefs such as thoughts of sudden death)
4. A disruption of usual or desirable functioning:

These characteristics are used to define anxiety disorders in the common diagnostic systems employed in psychiatry: the World Health Organization's International Classification of Disorders (ICD–10) and the American Psychiatric Association's Diagnostic and Statistical Manual (DSM-IV). In practice, these criteria may be difficult to apply to cancer patients.

It is difficult to judge when anxiety is disproportionate to the threat of cancer, since the disease is always associated with some real threat. The level of anxiety must be judged against the proximity of threat. For example, it is normal to experience considerable anxiety for a period of 7–10 days after receiving bad news (Holland, 1989), but as the degree of real threat varies throughout the history of the cancer, so therefore do levels of normal anxiety. In certain situations too little anxiety may be as problematic for adaptation as too much, and so while thoughts about recurrence and death will be natural early after diagnosis or relapse, they are not so during a long remission, with the point of transition being difficult to ascertain.

While the duration of symptoms is usually important in distinguishing abnormal anxiety the natural history of anxiety in oncology is uncertain, so this criterion is also difficult to apply. This may be because a range of definitions of abnormal anxiety have been used, and because anxiety is often labile and situational, making the onset of an episode difficult to define. Persistent anxiety may be identified quite early after diagnosis of cancer: anxiety which persists only 3 weeks after a 'bad-news consultation' is highly predictive of anxiety 6 months later (Nordin and Glimelius, 1999). The prevalence of anxiety problems after a cancer diagnosis falls over the following years (Fallowfield et al, 1994), but may not return to population levels even with curative treatment (Loge et al, 1997).

Unacceptable symptoms and disruption in functioning are often at least as useful in defining pathological anxiety in a cancer patient as other criteria. Intrusive and unpleasant anxious thoughts, often involving recurrence of disease, death, or disability, can cause considerable disruption in concentration, decision-making, sleep, and social functioning. Consequent behaviours, such as avoidance, repetitive checking of health, and seeking reassurance for transient somatic symptoms, can be disruptive for the individual and their family.

*Received 22 May 2000*
*Revised 30 June 2000*
*Accepted 30 June 2000*

*Correspondence to:* DPH Stark

**Table 1** The anxiety disorders

| Diagnosis | World Health Organization criteria | Cardinal features | | |
|---|---|---|---|---|
| | **Somatic symptoms** | **Symptom pattern** | **Situation** | **Response** |
| Panic disorder | At least four of the following including one from a to d:<br>a. Palpitation, pounding heart or accelerated heart rate<br>b. Sweating<br>c. Trembling/shaking<br>d. Dry mouth (not due to medication)<br>e. Difficulty in breathing<br>f. Feeling of choking<br>g. Chest pain or discomfort<br>h. Nausea or abdominal discomfort<br>i. Feeling dizzy, unsteady, faint or light-headed<br>j. Feeling that objects are unreal or that the self is distant or not really there<br>k. Fear of losing of control, going crazy, passing out<br>l. Fear or dying<br>m. Hot flushes or cold chills<br>n. Numbness or tingling sensations | A discrete episode of intense fear or discomfort, with a crescendo pattern, starts abruptly and reaches a maximum within a few minutes | Many situations | Hurried exit |
| Anxious adjustment disorder | As for other anxiety disorders, but insufficient to fulfil criteria for those | Onset of symptoms within 1 month of an identifiable psychosocial stressor | Not specific | Not specific |
| Phobia | As for panic disorder | Not specific | Specific | Avoidance |
| Generalized Anxiety Disorder | As for panic disorder, with the following also included:<br>o. Muscle tension or aches and pains<br>p. Restlessness and inability to relax<br>q. Feeling keyed up, on edge or mentally tense<br>r. Sensation of difficulty swallowing/lump in the throat<br>s. Exaggerated response to minor surprises/being startled<br>t. Difficulty concentrating or 'mind going blank' because of anxiety or worry<br>u. Persistent irritability<br>v. Difficulty in getting to sleep because of worrying | A period of 6 months with prominent, tension, worry and feelings of apprehension about everyday problems | Most situations | Not specific |

Abnormal anxiety can be classified based upon its clinical features. In the standardized diagnostic systems there are four main types of anxiety disorder (Table 1). The anxious adjustment disorder represents a quantitatively excessive response to a stressful event. However, a single severe negative life-event also increases the risk of generalized anxiety disorder. A diagnosis of generalized anxiety disorder requires more symptoms than anxious adjustment disorder, although degree of disruption is not a distinguishing feature. Adjustment disorders therefore take an intermediate position between other anxiety disorders and normal anxiety. The anxiety in panic disorder has specific features: it builds up in a rapid crescendo, it occurs in many situations so anticipatory avoidance is not feasible, and it is usually associated with a rapid exit from the situation in which it occurs. By contrast with these three disorders, phobic anxiety only occurs in specific situations, that is, in the presence of provoking stimuli, so anticipatory avoidance is common. In cancer care, phobias may arise in relation to hospital and to treatments in particular.

Anxiety may also be present in association with depression. In physically healthy populations mixed anxiety and depressive disorders are commoner than anxiety disorders occurring alone (Jenkins et al, 1998). Such an overlap also occurs in cancer populations, and therefore it has been recommended that clinicians assessing anxiety always seek co-existent depression, as treatment for depression may resolve the anxiety (Wise and Rieck, 1993).

Distinctions between pathological and adaptive anxiety, and between different forms of anxiety, are of some importance. Anxiety in a setting of distress, not psychiatric disorder, may be amenable to supportive care, and the response to specialized therapy differs between the diagnoses (Ramirez, 1989).

### EXTENT AND IMPACT OF ANXIETY IN CANCER PATIENTS

The prevalence of anxiety disorders in the general population is reported as between 3% and 16% in the UK (Jenkins et al, 1998). The reported prevalence of anxiety problems in cancer patient populations varies widely between studies (Table 2), but in comparisons within the general population pathological anxiety is commoner in people with cancer than in those without any chronic medical condition (Wells et al, 1988).

In the general population, younger women are more prone to anxiety (Jenkins et al, 1998). However age, gender, marital status, social class and education are not associations consistently seen with anxiety in cancer patient populations. Perhaps when the stressor is more severe, degrees of susceptibility may become less important (Noyes et al, 1998).

Anxiety certainly can cause disruption, and be detrimental to quality of life. A pattern of association between anxiety and self-reported quality of life, particularly impaired social functioning, fatigue and physical impairment, has been demonstrated in cancer

*© 2000 Cancer Research Campaign*

**Table 2** The prevalence of abnormal anxiety in observational studies of cancer patient

| Reference | Prevalence | Comments |
|---|---|---|
| Lee et al, 1992 | Anxiety 'cases': 17.8–6.5% over 12 months | Sample: Breast cancer, *n* = 159 |
| Fallowfield et al, 1994 | Anxiety disorders: 17–23% over 36 months | Sample: Breast cancer, *n* = 216 |
| Harrison and Maguire, 1994 | 2.3% generalized anxiety disorder<br>4.2% adjustment disorder with anxious mood | Sample: Mixed primaries, *n* = 520 |
| Kissane et al, 1998 | 1.7% generalized anxiety disorder<br>6.9% phobic disorder<br>1.3% panic disorder<br>3.9% adjustment disorder with anxious mood | Sample: Breast cancer, *n* = 303 |

The studies listed are restricted to those with sample sizes over 100, since 1990, where anxiety is measured by semi-structured interview, using International Classification of Disease or Diagnostic and Statistical Manual criteria care (Aass et al, 1997). Caution needs to be exercised in interpreting these associations, as there are several conflicting explanations for them, as to both the causal direction of the association and the explanation for the pattern.

The causal direction of this association might be that some disease-related symptoms are more anxiety-provoking than others. However, alternatively, physical symptoms identified in quality of life questionnaires may be due to anxiety rather than directly to cancer or its treatment. Forester et al (1993) illustrated that anxiety can contribute to anorexia, nausea, vomiting and fatigue in cancer patients, when they demonstrated reduction in these physical symptoms after psychotherapy.

Reporting of experience is not like playing back a tape-recording; individuals have to have noticed what took place, and to recall it, in order to reconstruct events. In anxious individuals this process may be systematically biased, favouring attention to, and possibly recall of, threatening experiences (Mogg et al, 1995). When we ask patients to assess their symptoms and functional status, particularly under the duress of an outpatient appointment, those problems which are threatening may be selectively reported.

Another perspective is that anxiety may influence the quantity of all symptoms that are recalled. For example, anxiety is associated with increased tamoxifen side-effects during breast cancer treatment, apparently due to the recall of more actual side-effects (Cameron et al, 1998).

The relation of anxiety to reported quality of life is of importance, as responses which reflect situational anxiety present in the outpatient clinic may not represent the patients' problems at other times.

Anxiety may also have consequences for the provision of cancer care. Psychiatric illnesses including anxiety, when present with physical illness, increase the resources used to manage that physical illness, independent of resource used in psychiatric care (House, 1996). Discharge from hospital cancer care to general practice is more difficult in the presence of anxiety (Thomas et al, 1997), perhaps because of greater severity of physical illness in this group or due to reluctance from the anxious patient.

### WHAT CAUSES ANXIETY – CANCER AS THREAT

### The disease

The idea of cancer is threatening, such that experiments which require anxiety-generating words may use the word cancer as a stimulus (Mogg et al, 1995). Anxiety levels are high soon after the onset of cancer symptoms, during investigation and diagnosis, but many people adapt over time (Fallowfield et al, 1994).

Anxiety appears to increase as illness progresses, such that more extensive disease is associated with higher prevalence of anxiety in the majority of studies (Noyes et al, 1998). However, such covariation of anxiety and progression of disease may not be found when analysis is controlled for physical disability (Schag and Heinrich, 1989; Aass et al, 1997).

As well as disability, other disease manifestations such as pain are associated with anxiety, although the causal direction is uncertain. For example, group therapy may improve breast cancer pain the extent of the improvement correlating with anxiety reduction (Spiegel and Bloom, 1983).

### The treatment

Cancer treatment involves a mixture of positive and negative implications, with unpleasantness and threat from the process and the hope of relief from the illness or its symptoms working in opposite directions.

In general, high levels of anxiety develop before surgery and abate afterwards, implying that the threat of surgery is viewed as short-term. Oncological surgery appears to be associated with a similar pattern. When Thomas et al (1987) measured psychological morbidity after colostomy, they found no difference in anxiety between patients who had the operation for malignant or benign disease. This suggests it is not the malignant element but the surgery itself that is relevant.

Chemotherapy and radiotherapy are associated with anxiety but the context is important. Toxicity of chemotherapy co-varies with anxiety, in a multivariate analysis controlled for progression of disease and performance status (Schag and Heinrich, 1989). Toxicity can thus represent a threat at the time it is occurring. However, for patients with inoperable lung cancer the highest level of anxiety is seen in patients not receiving chemotherapy or radiotherapy (Hughes, 1987); perhaps because no treatment is interpreted as meaning deterioration in health is unavoidable. In a randomized controlled trial of adjuvant chemotherapy against observation in early breast cancer, patients in the two arms had the same anxiety levels (Cassileth et al, 1986). In this setting, chemotherapy appears not to cause more perceived threat than observation, or alternatively both had the potential to be threatening to some individuals.

Patients undergoing serial intracavitary radiotherapy for gynaecological cancer experience high levels of anxiety, beginning prior to the intervention, but these do not rapidly dissipate or habituate

© 2000 Cancer Research Campaign for later treatments (Andersen et al, 1984). The threatening element is persistent, possibly because of the serial element of the therapy, or the intimate nature of the intervention. Patients ending radiotherapy experience a rise in anxiety; perhaps the interpretation is the loss of a perceived protective effect (Holland, 1989).

Other specific elements of the cancer patients' experience influence anxiety Magnetic resonance scans are associated with anxiety in up to a third of patients, particularly those with prior history of panic (Melendez and McCrank, 1993). It might be assumed that the problem is the investigation, as the subject is highly enclosed. While this may be the explanation, during CT scans the major concern of patients is with the disease not the scan process (Peteet et al, 1992). Each can be threatening, but not necessarily for the same reasons.

Anxiety symptoms in cancer care may be an organic phenomenon, often pharmacological. Some organic causes are well known, such as corticosteroids. Others are less so, for example there can be an early rise in anxiety associated with the antidepressant fluoxetine, and the adverse effects of biological therapies such as interferon alpha include psychiatric syndromes (Trask et al, 2000).

While it is clear that cancer is a threat to future health and to life, it is not a cause of disabling anxiety in the majority of patients. It does not seem that particular parts of the process of illness, investigation and treatment are predictably making most patients anxious. Instead it may be necessary to explore the interpretations and meanings attached to events for the individual. This was observed by Lazarus, who characterized patient responses to illness in terms of the emotional elements of events; fear, anger, or sadness, and the meaning of the cancer; its identity, time-course, consequences, cause, and controllability (Lazarus, 1993). Both these emotional and cognitive elements contribute to assessing the threat. The meaning of an event may then be the crux of why an individual is anxious, and that meaning may not be what is immediately apparent to the clinician, and be specific to individual patients and their circumstances.

### THE MANAGEMENT OF ANXIETY WITHIN ONCOLOGY

Often oncologists and cancer care nurses will be responsible for the identification of patients who have problems with anxiety. Screening has been used in an attempt to improve detection of psychiatric morbidity, including anxiety. It is acceptable to patients and amenable to some automation (Velikova et al, 1999). There are various screening questionnaires (Table 3), each with their individual advantages and disadvantages (Hall et al, 1999; Trask et al, 2000). There is no brief screening questionnaire which specifically measures anxiety. The Hospital Anxiety and Depression Scale, the General Health Questionnaire, and many quality of life instruments include anxiety items, but it is uncertain to what extent they distinguish anxiety from non-specific distress, unless they are followed by clinical assessment. There are few direct comparisons of the efficacy of these in the general oncology outpatient population. Screening can be valuable however, and there is a trend for greater efficacy of psychological interventions in screened populations than for those not screened (Sheard and Maguire, 1999).

Having identified that an individual has problems with anxiety, there is a need to intervene either within cancer care or by referring for specialist treatment. There are several pharmacological interventions of demonstrated efficacy (Table 4). There is a wide range of drug interventions available but have been few studies comparing their efficacy in cancer patients, and therefore decisions are often based upon the urgency for treatment effect to begin, clinical experience with individual agents, and toxicity profiles. This area has been recently reviewed (Kerrihard et al, 1999).

Psychological treatments for cancer patients with anxiety or depression have recently been the subject of a meta-analysis of published and unpublished studies (Sheard and Maguire, 1999). This reiterated the effectiveness for anxious cancer patients of individual and group therapies, and of educational interventions as well as relaxation, when delivered by mental health specialists. Notably there was no difference in effectiveness of such treatments between patients with metastatic and those with better-prognosis disease. However, treatments needed an experienced therapist and were more likely to be beneficial the longer they took, which could have substantial consequences for provision of services.

Involvement of a mental health professional is not a useful option for many anxious cancer patients. The range of available services is often limited, and depends upon the selection of cases appropriate for referral. In addition, not all patients are prepared to

**Table 3** Some screening tools commonly used to assess anxiety by patient self-report

| Measure | Advantages to the oncologist | Disadvantages to the oncologist |
|---|---|---|
| Hospital Anxiety and Depression Scale (Zigmond and Snaith, 1983) | Excludes somatic symptoms of disease<br>Brevity (14 items in all, 7 concerning anxiety)<br>Widespread use in cancer and other physical illnesses.<br>More effective than many other instruments: at a cut-off of 7 the anxiety scale missed 28% of the individuals with morbid anxiety at interview, and 21% of those without morbid anxiety were found to have scores above the threshold (Hall et al, 1999).<br>Use as a screen and a measure longitudinally of progress (Kissane et al, 1998; Fallowfield et al, 1994) | Used alone is poor at detecting depression. The depression subscale missed at least 62% of cases with depression at interview (Hall et al, 1999) |
| State-Trait Anxiety Inventory (Spielberger, 1983) | Specific to anxiety<br>Use as a screen and a measure longitudinally | Used alone is not detecting depression<br>Longer (20–40 items) than many self-report screening questionnaires |
| General Health Questionnaire, (Goldberg et al, 1997) | Brevity (12-item version)<br>Excludes somatic symptoms of disease<br>Use as a screen and a measure longitudinally | Screening efficacy in oncology outpatients remains uncertain. |

 *© 2000 Cancer Research Campaign*

**Table 4** Pharmacological interventions in anxious cancer patients

| Intervention | Comments |
|---|---|
| Beta-blockers | Help to control unpleasant palpitation and tremor, but not anxiety itself |
| Tricyclic antidepressant, such as imipramine | Anxiolytic effect is slow in onset (weeks)<br>Not dependency inducing<br>Useful in panic disorder, or in anxiety with depression<br>Anticholinergic effects can be ameliorated by a low starting dose |
| Selective serotonin reuptake inhibitors, such as paroxetine | Less toxicity than tricyclic antidepressants, particularly anti-cholinergic effects |
| Short-acting benzodiazepine, such as alprazolam | All benzodiazepines are potentially dependency inducing and therefore should only be used to cover periods where an identified stressor will end after a short period (days)<br>Rapid onset of effect, but problems may recur on withdrawal<br>Less likely than long-acting benzodiazepines to accumulate in liver disease |
| Long-acting benzodiazepine, such as diazepam | Useful to manage recurrent symptoms after a course of short-acting benzodiazepine |
| Neuroleptic, such as haloperidol | Useful adjunct to benzodiazepines<br>Less respiratory depression than benzodiazepines<br>Not dependency inducing.<br>Avoid long-term use because of risk of tardive dyskinesia |

From Kerrihard et al, 1999

accept referral, and so there is a need for the management of many of these problems to take place within cancer care.

Giving information is often the first step in helping anxious patients. While information needs to be tailored to the wishes of the individual (Leydon et al, 2000) many patients in the UK want more information about their cancer. Poor or incomplete information can generate mistrust of medical staff. Patients are not deprived of hope by information; perhaps they still consider themselves within a group of individuals likely to respond to treatment whatever their understanding of the odds of that happening. Patients do not necessarily ask for the further information they want, so many retain lay perceptions that are ‘worse than the facts’ (Fallowfield, 1997). Knowledge may therefore achieve reduction in anxiety, when it is tailored to an understanding of the meaning of events to the patient.

Patient choice in treatment appeared beneficial in early studies, however when this work was repeated in a larger sample a benefit was not demonstrated for choice. Instead those who adapted best were patients under the care of surgeons who offer choice where technically feasible, whether to that patient or not, and those who were satisfied with the information received about the illness and treatment. This points to the role of communication skills (Fallowfield et al, 1994).

Effective communication skills are central to information giving, with substantial correlation between anxiety and poor communication with the medical team (Schag and Heinrich, 1989). Workshops encouraging open questioning, psychological issues, empathy and summarizing, while discouraging reassurance, ‘advice mode’, and leading questions have achieved enduring change and greater disclosure of patients’ psychosocial problems (Maguire, 1995).

Over and above these general approaches the oncologist needs to be alert to specific difficulties in communication with anxious cancer patients.

Anxious individuals experience and report more symptoms related to their cancer and its treatment than others. Oncologists are often asked to reassure anxious patients that their experienced symptoms are not due to worsening of the cancer. However, even in the absence of progression of the cancer after appropriate investigation, well-meaning *simple* reassurance may inadvertently *worsen* anxiety. Anxiety may be temporarily reduced, such that it may appear during the consultation that anxiety has been effectively managed, but often it rapidly returns. The implication may be that the cause of the anxiety is not removed by many consultations, although this is the aim (Lucock et al, 1997).

A cognitive model of this problem has been suggested, which has the potential advantage of allowing oncologists to intervene specifically to help some anxious patients. The model identifies a group of beliefs and behaviours characteristic of patients who are anxious about their health:

- *Beliefs*: Tending to interpret everyday bodily symptoms as indicative of serious disease
- *Concerns*: Health worry and preoccupation, fear of serious illness, and of death. This can be intrusive and difficult to control
- *Behaviours*: Reassurance-seeking, including seeking medical consultations.

While this model is derived from work on extreme levels of psychological morbidity such as hypochondriasis, it applies in general medical settings and general practice (Lucock et al, 1997; Conroy et al, 1999). After a cancer diagnosis, patients learn to monitor their bodies for symptoms of relapse, and interpret their symptoms. Health anxiety may be seen as a maladaptive pattern of such monitoring.

The anxious individual is highly vigilant to threatening stimuli, but when they notice them they employ ‘quick-fix’ techniques to reduce the emotion this causes (Lucock et al, 1997). Seeking expert advice about non-specific symptoms when living with cancer or a previously treated cancer is of course not necessarily maladaptive. Reassurance-seeking behaviour is, however, maladaptive if it is out of proportion to the symptoms present. It may be so also if it becomes an enduring coping pattern, where reassurance reduces the anxiety only temporarily and then reassurance-seeking is used again, or increasingly, perpetuating the anxiety rather than developing more constructive responses.

To intervene more effectively an oncologist may need a distinct approach to improving communication with anxious cancer

© 2000 Cancer Research Campaign 

patients. They could go beyond eliciting symptoms and concerns and offering reassurance, and discuss the patients' *interpretation* of their symptoms. They might discuss the nature of symptoms in general, outlining and perhaps providing a simple checklist of sinister and non-sinister problems, to provide education that the patient could apply to reduce their own anxiety when future symptoms occur. This distinct approach has the potential to reduce the anxiety of some cancer patients.

### CONCLUSIONS

Morbid anxiety in the setting of cancer care can be a problem in its own right. It can be difficult to use all the current research criteria to define when anxiety is pathological, because they depend upon a subjective judgement as to the extent of actual threat, and rigorous studies defining the natural history of normal or adaptive anxiety are incomplete. However, the presence of severe or disruptive symptoms such as panic attacks can be diagnostic regardless of context.

It can be helpful to determine which form of anxiety problem is present for a specialist pharmacological or psychological intervention, but this form of management is neither universally available, nor acceptable to all patients. Oncologists may need to develop their own approach to anxious patients. There are several suggestions from the literature in this area:

1. It is helpful to explore the meaning patients attach to events. These are the basis of the perceived threat for the individual, and they may differ from the perceptions of clinicians.
2. Information and education are important in alleviating anxiety, even if the situation is difficult, as fears are often based upon incorrect information.
3. Communication can be particularly difficult with the anxious patient. It may be helpful to avoid responding with simple reassurance when the anxious patient raises a symptom. While this may result in a short-term dissipation of anxiety in the course of the consultation, it is often ineffective in durably resolving the patients' anxiety. Instead, eliciting concerns of the patient beyond a symptom itself, exploring and correcting the ways patients interpret the symptoms they experience, may be more likely to enduringly reduce anxiety.

### ACKNOWLEDGEMENT

Dr Stark is grateful for grant support from the St. James's and Seacroft NHS Trust Special Trustees.

### REFERENCES

Aass N, Fossa SD, Dahl AA and Moe TJ (1997) Prevalence of anxiety and depression in cancer patients seen at the Norwegian Radium Hospital. *Eur J Cancer* **33**: 1597–1604

Andersen BL, Karlsson JA, Anderson B and Tewfik HH (1984) Anxiety and cancer treatment: response to stressful radiotherapy. *Health Psychol* **3**: 535–551

Cameron LD, Leventhal H and Love RR (1998) Trait anxiety, symptom perceptions, and illness-related responses among women with breast cancer in remission during a tamoxifen clinical trial. *Health Psychol* **17**: 459–469

Cassileth BR, Knuiman MW, Abeloff MD, Falkson G, Ezdinli EZ and Mehta CR (1986) Anxiety levels in patients randomized to adjuvant therapy versus observation for early breast cancer. *J Clin Oncol* **4**: 972–974

Conroy RM, Smyth O, Siriwardena R and Fernandes P (1999) Health anxiety and characteristics of self-initiated general practitioner consultations. *J Psychosom Res* **46**: 45–50

Fallowfield L (1997) Truth sometimes hurts but deceit hurts more. *Ann NY Acad Sci USA* **809**: 525–536

Fallowfield LJ, Hall A, Maguire P, Baum M and A'Hern RP (1994) Psychological effects of being offered choice of surgery for breast cancer. *BMJ* **309**: 448–448

Forester B, Kornfeld DS, Fleiss JL and Thompson S (1993). Group psychotherapy during radiotherapy: effects on emotional and physical distress. *Am J Psychiatry* **150**: 1700–1706

Goldberg DP, Gater R, Sartorius N, Ustun TB, Piccinelli M, Gureje O and Rutter C (1997) The validity of two versions of the GHQ in the WHO study of mental illness in general health care. *Psychol Med* **27**: 191–197

Hall A, Ahern R and Fallowfield L (1999) Are we using appropriate self-report questionnaires for detecting anxiety and depression in women with early breast cancer? *Eur J Cancer* **35**: 79–85

Harrison J and Maguire P (1994) Predictors of psychiatric morbidity in cancer patients. *Br J Psychiatry* **165**: 593–598

Holland JC (1989) Anxiety and cancer: the patient and the family. *J Clin Psychiatry* **50 Suppl**: 20–5, 20–25

House A (1996) Psychiatric disorders, inappropriate health service utilisation and the role of consultation-liaison psychiatry. *J Psychosom Res* **40**: 443–443

Hughes JE (1987) Psychological and social consequences of cancer. *Cancer Surv* **6**: 455–475

Jenkins R, Bebbington P, Brugha TS, Farrell M, Lewis G and Meltzer H (1998) British psychiatric morbidity survey. *Br J Psychiatry* **173**: 4–7

Kerrihard T, Breitbart W, Dent R and Strout D (1999) Anxiety in patients with cancer and human immunodeficiency virus. *Semin Clin Neuropsychiatry* **4**: 114–132

Kissane DW, Clarke DM, Ikin J, Bloch S, Smith GC, Vitetta L and McKenzie DP (1998) Psychological morbidity and quality of life in Australian women with early-stage breast cancer: a cross-sectional survey. *Med J Aust* **169**: 192–196

Lazarus RS (1993) Coping theory and research – past, present, and future. *Psychosom Med* **55**: 234–247

Lee MS, Love SB, Mitchell JB, Parker EM, Rubens RD, Watson JP, Fentiman IS and Hayward JL (1992) Mastectomy or conservation for early breast cancer: psychological morbidity. *Eur J Cancer* **28A**: 1340–1344

Leydon G, Boulton M, Moynihan C, Jones A, Mossman J, Boudioni M and McPherson K (2000) Cancer patients' information needs and information seeking behaviour: in-depth interview study. *BMJ* **320**: 909–913

Loge JH, Abrahamsen AF, Ekeberg O, Hannisdal E and Kaasa S (1997) Psychological distress after cancer cure: a survey of 459 Hodgkin's disease survivors. *Br J Cancer* **76**: 791–796

Lucock MP, Morley S, White C and Peake MD (1997) Responses of consecutive patients to reassurance after gastroscopy: results of self administered questionnaire survey. *BMJ* **315**: 572–575

Maguire P (1995) Psychological interventions to reduce affective disorders in cancer patients – research priorities. *Psycho-Oncology* **4**: 113–119

Melendez JC and McCrank E (1993) Anxiety-related reactions associated with magnetic resonance imaging examinations. *JAMA* **270**: 745–747

Mogg K, Bradley BP and Williams R (1995) Attentional bias in anxiety and depression – the role of awareness. *Br J Clin Psychol* **34**: 17–36

Nordin K and Glimelius B (1999) Predicting delayed anxiety and depression in patients with gastrointestinal cancer. *Br J Cancer* **79**: 525–529

Noyes RJ, Holt C and Massie M (1998) Anxiety disorders. In *Psycho-oncology*, Holland JC (ed) pp 548–563. New York: Oxford University Press

Peteet JR, Stomper PC, Ross DM, Cotton V, Truesdell P and Moczynski W (1992) Emotional support for patients with cancer who are undergoing CT: semistructured interviews of patients at a cancer institute. *Radiology* **182**: 99–102

Ramirez AJ (1989) Liaison psychiatry in a breast cancer unit. *J R Soc Med* **82**: 15–17

Schag CA and Heinrich RL (1989) Anxiety in medical situations: adult cancer patients. *J Clin Psychol* **45**: 20–27

Sheard T and Maguire P (1999) The effect of psychological interventions on anxiety and depression in cancer patients: results of two meta analyses. *Br J Cancer* **80**: 1770–1780

Spiegel D and Bloom JR (1983) Group therapy and hypnosis reduce metastatic breast carcinoma pain. *Psychosom Med* **45**: 333–339

Spielberger CD (1983) *The Manual for the State-Trait Anxiety Inventory.* Palo Alto: Consulting Psychological Press

Thomas C, Madden F and Jehu D (1987) Psychological effects of stomas – I. Psychosocial morbidity one year after surgery. *J Psychosom Res* **31**:311–316

Thomas SF, Glynne-Jones R, Chait I and Marks DF (1997) Anxiety in long-term cancer survivors influences the acceptability of planned discharge from follow-up. *Psycho-Oncology* **6**: 190–196

 *© 2000 Cancer Research Campaign*

Trask PC, Esper P, Riba M and Redman B (2000) Psychiatric side effects of interferon therapy: prevalence, proposed mechanisms, and future directions. *J Clin Oncol* **18**: 2316–2326

Velikova G, Wright EP, Smith AB, Cull A, Gould A, Forman D, Perren T, Stead M, Brown J and Selby PJ (1999) Automated collection of quality-of-life data: a comparison of paper and computer touch-screen questionnaires. *J Clin Oncol* **17**: 998–1007

Wells KB, Golding JM and Burnam MA (1988) Psychiatric disorder in a sample of the general population with and without chronic medical conditions. *Am J Psychiatry* **145**: 976–981

Wise MG and Rieck SO (1993) Diagnostic considerations and treatment approaches to underlying anxiety in the medically ill. *J Clin Psychiatry* **54 (Suppl)** 22–26

Zigmond AS and Snaith RP (1983) The hospital anxiety and depression scale. *Acta Psychiatr Scand* **67**: 361–370

*© 2000 Cancer Research Campaign*
